# Prospects for Anti-Tumor Mechanism and Potential Clinical Application Based on Glutathione Peroxidase 4 Mediated Ferroptosis

**DOI:** 10.3390/ijms24021607

**Published:** 2023-01-13

**Authors:** Mingliang Chen, Zhihao Shi, Yuqiu Sun, Haoran Ning, Xinyu Gu, Lei Zhang

**Affiliations:** 1 School of Basic Medical Science, Henan University, Kaifeng 475004, China; 2 School of Clinical Medicine, Henan University, Kaifeng 475004, China

**Keywords:** ferroptosis, glutathione peroxidase 4, selenocysteine, cancer, anti-tumor

## Abstract

Ferroptosis, characterized by excessive iron accumulation and lipid peroxidation, is a novel form of iron-dependent cell death, which is morphologically, genetically, and biochemically distinct from other known cell death types, such as apoptosis, necrosis, and autophagy. Emerging evidence shows that glutathione peroxidase 4 (GPX4), a critical core regulator of ferroptosis, plays an essential role in protecting cells from ferroptosis by removing the product of iron-dependent lipid peroxidation. The fast-growing studies on ferroptosis in cancer have boosted a perspective on its use in cancer therapeutics. In addition, significant progress has been made in researching and developing tumor therapeutic drugs targeting GPX4 based on ferroptosis, especially in acquired drug resistance. Selenium modulates GPX4-mediated ferroptosis, and its existing form, selenocysteine (Sec), is the active center of GPX4. This review explored the structure and function of GPX4, with the overarching goal of revealing its mechanism and potential application in tumor therapy through regulating ferroptosis. A deeper understanding of the mechanism and application of GPX4-mediated ferroptosis in cancer therapy will provide new strategies for the research and development of antitumor drugs.

## 1. Introduction

According to recent cancer statistics, there were approximately 19.3 million new cases in 2020 and about 10 million cancer-related deaths worldwide [1]. Meanwhile, the cancer burden is expected to rise significantly due to the aging global population. It is worth noting that cancer treatment is hampered by the complexity of the disease, its rapid development, and its difficult-to-control nature. To date, surgery, radiotherapy, and chemotherapy used in single or combined therapy are still the traditional treatment means; however, their curative effects are accompanied by severe limitations and detrimental side effects. In recent years, significant advances have been made in cancer therapies, including stem cell therapies, targeted therapies, nano therapies, ferroptosis-related therapies, and their synergistic combination with conventional schedules. However, apoptosis resistance of tumor cells is becoming obvious as an unavoidable weakness of these therapies, and a major reason for the failure of same. Since ferroptosis was formally defined, numerous studies have suggested that it plays an essential role in the progression of various diseases, especially in cancer and neurodegenerative diseases [2,3,4,5].

Ferroptosis, formally defined by Dixon et al. [6,7], is a new type of cell death that differs from apoptosis, necrosis, and autophagy and is characterized by a large amount of iron-related lipid peroxidation and accumulation of reactive oxygen species (ROS) [8]. GPX4, an antioxidant enzyme belonging to the glutathione peroxidase family (GPXs), is a kind of selenoprotein containing selenocysteine (Sec) that is affected by selenium in many ways and in many different situations [9]. Many studies have shown that GPX4 is one of the inhibitors of ferroptosis through scavenging lipid peroxides. Thus, it can be used as a reference marker to recognize ferroptosis in cells [8]. Inactivation of GPX4 leads to oxidative imbalance and destruction of the membrane structure by lipid peroxides, thereby triggering ferroptosis [10]. Therefore, given its unique mechanism, the role of GPX4 in ferroptosis has recently become a new and popular research field, on the basis of ferroptosis in cancer therapy [11]. Based on the results of our wide research in PubMed, Web of Science, and FerrDB databases, This review focus on the structure, expression regulation, and function of GPX4 in ferroptosis and the relationship between GPX4 and its influence on ferroptosis in cancer therapy. Moreover, the paper summarizes and analyzes the research progress on its clinical application and prospects for its development.

## 2. The Structure of GPX4 and GPX Family

GPX4, also known as phospholipid peroxide hydro glutathione peroxidase (PHGPx), is a vital oxidoreductase belonging to the GPX family that has a critical role in controlling levels of ROS. As the fourth GPX member containing selenium, the *GPX4* gene is located at 19p3.3 in the human genome and consists of seven exons translated into a polypeptide chain of 170 amino acid residues with a theoretical molecular weight of 19 kDa [12,13]. GPX4 is composed of a thioredoxin motif of four solvent-exposed alpha helices and seven beta strands. The active sites of GPX4 include three residuals: selenocysteine (U46), glutamine (Q81), and tryptophan (W136), all of which are likely to make GPX4 less functional [14,15]. A previous study indicated that when selenocysteine is mutated to cysteine, GPX4 activity is reduced by 90%, suggesting that the Sec residue is necessary to maintain the full activity of GPX4. 

There are also other members of the GPX4 family in mammals, including GPX1-GPX8, which is divided into three parts according to similarity and differences in amino acid sequences: GPX1-GPX3, GPX5-GPX6, and GPX4 and GPX7-GPX8. GPX1-GPX4 and GPX6 are selenium and contain Sec, which is the necessary active site [16]. Notably, GPX1-GPX4 can prevent oxidation attacks and inhibit inflammation, but the function of GPX6 is not clear [17]. GPX1 and GPX4 mainly inhibit the phosphate cascade by preventing the inactivation of hydrogen peroxide or lipid hydrogen peroxide to phosphatase. Although GPX2 regulates the regenerative balance of intestinal cells and inhibits inflammation-induced bowel cancer, it also promotes the growth of diagnosed cancers. On the other hand, GPX3 is considered a tumor inhibitor. The remaining members of the GPX family have cysteine as the active site. A recent study indicated that GPX4 and GPX5 play a new role in male fertility. GPX7 reacts with GPX8 and protein isomerase and plays a unique role in oxidizing protein folding in the internal mesh [18,19]. Unlike other GPX family members, GPX4 can effectively remove membrane lipid hydrogen peroxide products to resist lipid peroxide reaction, a fundamental antioxidant system in organisms to maintain lipid redox stability, thereby preventing ferroptosis [20].

## 3. Regulation of GPX4 Expression

### 3.1. Genetic Transcription and Transcriptional Regulation of GPX4

Generally, GPX4 has three different basic isoforms [cytosolic (c-GPX4), mitochondrial (m-GPX4), and nuclear (n-GPX4)] originating from different parts of the same *GPX4* gene [21]. C-GPX4 starts from the first exon’s second 59-ATG (c-ATG), whereas m-GPX4 uses the first 59-ATG (m-ATG) of the first exon. In contrast to c-GPX4 and m-GPX4, n-GPX4 is predominantly synthesized during late spermatogenesis. Its expression is associated with the transcription of an alternative exon (exon 1B localized in the first intron of the *GPX4* gene) that encodes for a nuclear targeting sequence [22]. It should be noted that c-GPX4 is universally expressed in most mammalian cells, m-GPX4 mostly appears in spermatoid cells, and n-GPX4 is expressed in late spermatocytes [23,24]. Different positions and stages of expression suggest the activation of transcription factors Sp1 and NF-Y, which influence the expression of c-GPX4 and m-GPX4. Combination of the activated cAMP-response element modulator-tau to the 5′-flanking region of exon 1B has also been included; hence, c-n-GPX4 and m-n-GPX4 exhibit different expression kinetics [25,26]. Similarly, a previous study confirmed that CRE binds the transcription factor cAMP-response element modulator (CREM), which mediates the precise expression of n-GPX4 [27]. Moreover, Alim et al. [28] conducted experiments in intracerebral hemorrhage (ICH) models and found that pharmacological selenium augments the *GPX4* gene in this transcriptional program through coordinated activated transcription factors: TFAP2c and Sp1, which is consistent with the previous conclusion [28].

### 3.2. The Regulation of GPX4’s Post-Transcriptional and Translational Level

Co-translational incorporation of Sec is the focal point of research on post-transcriptional elements included in GPX4 expression regulation. Sec, a stop signal in translation, is built in by the ribosome using a UGA codon. The 3′ untranslated regions (UTRs) of selenoprotein mRNAs contain a secondary structure called the Sec insertion sequence (SECIS) element, which controls the UGA to keep off the formation of truncated proteins so that it can prevent premature translational termination [29,30,31,32,33,34]. A later study revealed that the interaction between SECIS and SECIS binding protein 2 (SBP2) is a vital nodal point for the metabolic balance between selenium and selenoproteins, and GPX4 has specific binding appeal to SBP2 in cells [35]. To sum up, the expression of GPX4 is associated with the executive force of selenium. Ufer et al. [21] found that the guanine-rich sequence-binding factor 1 (Grsf1) upregulates GPX4 expression, which clarified the translation mechanism in GPX4 expression. Grsf1 binds to a particular target sequence in the 5′ UTR of *m-GPX4* mRNA, thereby upregulating UTR-dependent reporter gene expression and recruiting *m-GPX4* mRNA to translationally active polysome fractions, which speeds up the translation. Grsf1 and m-GPX4 are co-expressed during embryonic brain development, and functional knockdown (siRNA) of Grsf1 causes the depression of embryonic GPX4 expression. *CircIL4R*, a kind of circular RNA (circRNA), can regulate gene expression as “miRNA sponges” [36]. Xu et al. [37] reported that *circIL4R* positively influenced GPX4 regulation via sponging microRNA-541-3p (*miR-541-3p*) in hepatocellular carcinoma cells, thereby influencing GPX4 expression at both mRNA and protein levels. 

### 3.3. Regulation after Translation

Modification at the protein level is the primary regulatory mechanism for maintaining the stability of GPX4 protein. Autophagy, a widespread process in the body, is shown to be an important cellular mechanism for amino acid utility. Lee et al. [38] found that the lipid peroxidation product, 4-hydroxy-2-nonenal (HNE) increased in LAMP2 knockdown cells. Therefore, we can speculate the possible direct link between autophagy and GPX4 protein. The heat shock 70 kDa protein 5 (HSPA5, also termed GRP78 or BIP) is a member of the molecular chaperones primarily expressed in the endoplasmic reticulum (ER) [39]. Studies have revealed that it can promote cell survival under conditions of ER stress, thus acting as an essential component of the unfolded protein response [40]. Zhu et al. [41] found that increased HSPA5 expression causes direct inhibition of GPX4 protein degradation in human PDAC cells. The combination of GPX4 to HSPA5 is mainly regulated by U46. The regulation of GPX4 protein may be induced by oxidative phosphorylation (OXPHOS) dysfunction, but the specific effects and mechanisms have not yet been fully explored [42]. The factors described above have been shown to influence the regulation of GPX4 expression, which suggests that we can maintain the physiological function of GPX4. At the same time, it is evident that ferroptosis can be influenced by regulating GPX4 at a specific target [43].

## 4. Selenocysteineis Significant to GPX4, Not Selenium Treatment

Sec, the genetic code’s 21st amino acid, is one atom different from cysteine (i.e., selenium replacing S in thiol) [44], which significantly contributes to improving the catalytic activity of thiol oxidoreductases [45]. One of the essential pathways for regulating the biosynthesis of Sec-bearing-GPX4 is the mevalonate (MVA) pathway. Considering that the genetic code of Sec is UGA, a code shared with the termination codon, a Sec tRNA (tRNA(Sec)) transporter is required to import Sec into GPX4 specifically [9]. Notably, tRNA(Sec) is a vital component of the mechanism and controls the biosynthesis of the 25 selenoproteins in the human body [46]. It contains isopentenyladenosine and can decode the genetic code of Sec and accurately insert Sec into the corresponding protein [47]. However, tRNA-isopentenyl transferase is required for the maturation of Sec tRNA to catalyze the transfer of the isopentene group of isopentenyl pyrophosphate (IPP) to the specific adenine sites of Sec tRNA precursors [48]. Statins, which inhibit the MVA pathway, have been shown to impede tRNA(Sec) maturation and biosynthesis of GPX4 in cell culture [46,48]. Therefore, further studies into MVA-pathway-regulated ferroptosis should be conducted to explore the effect of targeting the MVA pathway in human diseases like cancer. 

However, the actual advantage of Sec-GPX4 over Cys-GPX4 is still unknown. Yu et al. [49] reported that a mutant GPX4 catalyst with noncatalytic Sec residues substituted for Cys showed significantly more deficiency in Escherichia coli than in natural Sec-GPX4. Mannes et al. [50] found that wild-type GPX4 and its Sec/Cys mutant successfully rescued apoptosis of GPX4-disrupted cells, whereas Sec/Ser mutants failed. The Doxycycline-inducible expression test further confirmed that the wild-type GPX4 was more efficient. However, Cys substitution of Sec in different selenoproteins depends on selenium utilization of cells [51]. Ingold et al. [16] conducted a study on the ability of GPX4^Cys/Cys^ cells to treat peroxidation. Intriguingly, they observed an unforeseen sensitivity to cell death induced by peroxides, whereas wild-type cells retained a relatively high activity. Thus, although further studies on the actual influence on GPX4 function by selenium are required, we believe that the shortage of selenium may have a significant impact on GPX4 antioxidant activity. However, it should be noted that excessive selenium supplement is toxic, with consequences involving decreased GPX4 activity [52] and increased risk of type-2 diabetes [53].

It is well known that GPX4 is essential to spinal cord growth. GPX4 mutations in two families affected by Sedaghatian-type spondylometaphyseal dysplasia (SSMD) were found to cause shortened GPX4 variants and ultimately null versions [54]. SSMD is a neonatal lethal form of spondylometaphyseal dysplasia characterized by severe metaphyseal chondrodysplasia with mild limb shortening, platyspondyly, cardiac conduction defects, and central nervous system abnormalities [55]. Given that deletion of GPX4 in mice was found to be lethal in the early-embryonic stage, it is astounding that mutant patients still proceed to embryogenesis with a shortened, inactive form of GPX4 [56,57]. However, in an experiment conducted by Ingold et al. [16], mice bearing GPX4^Cys/Cys^ were born normally, but appeared to lose weight after 14–16 postnatal days and thus had to be sacrificed 18 days after birth. They were also observed to be suffering severe spontaneous seizures or hyperexcitable states. This may be attributed to an irreversible inactivation of GPX4 [54], whose selenium is deprived. However, further studies should be conducted to determine what makes the specific type of neurons dependent on selenium-containing GPX4.

The underlying mechanisms of selenium in cancer therapy have not yet been fully elucidated and are still under ongoing research. Some studies have demonstrated that they involve a range of effects, including proteomic and prooxidant activities, which eventually induce apoptosis, paraptosis, necrosis, and ferroptosis [58,59,60,61]. These findings imply that the mechanisms through which high-dose selenium treats cancer are motivated by the toxicological properties of selenium [62,63,64]. Therefore, further animal experiments and clinical trials on selenium compounds should be conducted to evaluate their safety.

According to Vinceti et al. [65], one of the two most well-known clinical trials to test the effect on cancer of selenium supplementation was launched at the Arizona Cancer Center in 1991, and a decreased incidence of prostate cancer was observed [66,67,68,69]. In contrast, another study conducted by the U.S. National Cancer Institute found a contradicting result, that the incidence of prostate cancer was increased [70]. It is worth mentioning that both trials had no satisfying results, with the former showing an excessive incidence of nonmelanoma skin cancer and had more collateral risks, whereas the latter was interrupted by the trial Data Safety Monitoring Committee in 2008 due to lack of efficacy and the excessive prostate cancer incidence [71,72]. Although two trials have some consistent findings like exceeded diabetes risks, arguments upon those results still exist [65,73]. Further research on selenium treatment to affect tumor therapy is rare. The trial research above did not prove any potential influence of selenium treatment on selenoproteins, including Sec and GPX4, in tumor therapy.

## 5. GPX4 and Lipid Peroxidation

It is well known that oxygen is necessary for many chemical reactions in cells and is involved in many metabolic pathways. The production of rich ATPs in aerobic metabolism is accompanied by the generation of ROS, a single electron reduction product of a class of oxygen in the body that is produced by electrons leaking out of the respiratory chain and consuming about 2% oxygen before they can be transferred to the end oxidase, including one-electron reduction product superoxide anion (O_2_^.−^), two-electron reduction product hydrogen peroxide (H_2_O_2_), three-electron reduction product hydroxyl radical (^.^OH), and nitric oxide. Notably, ROS is mainly produced in the mitochondrion due to the high oxygen environment and high reduction of the respiratory chain in the transition from state III to state IV, which causes a large number of electrons to leak out and reduce oxygen molecules. As an essential medium of transmitting the signal within the cell, excessive ROS production can lead to oxidative stress, ultimately resulting in cell death.

Mammalian cell membranes are mainly composed of phospholipids and sterols. Lipid peroxidation is a free radical chain reaction process, which occurs in three steps: the first step is a chain-induced reaction where lipid free radicals are produced after the active substance and the lipid oxidizing substance extract hydrogen atoms. The second step is the chain growth response. The lipid free radicals produced in the first step are extremely unstable and easily react with molecular oxygen to produce peroxide lipid free radicals. Therefore, they stimulate other lipid molecules into lipid free radicals and hydrogen peroxide, and thus the cycle is repeated. The third step is the chain termination reaction. At this point, enough free radicals and free radical reactions produce non-free radical substances, and the response terminates (but only if the concentration of the free radical species is high enough that the probability of collision between the two free radicals is high) [74]. However, not all lipids are just as easily oxidized. Specifically, polyunsaturated fatty acids are more easily oxidized than saturated fatty acids and monounsaturated fatty acids, and the greater the amount of bisallylic hydrogen atoms in polyunsaturated fatty acids, the easier it is to lose hydrogen atoms. In addition to phospholipids, cholesterol and other sterols on the cell membrane can be automatically oxidized. Notably, although the C-H bond in cholesterol is stronger than in polyunsaturated fatty acids, the higher cholesterol content in the membrane makes the lipid peroxidation reaction possible [14].

The reaction equations of lipid peroxidation:

L-H + R· → L· + R-H(1)

L· + O_2_ → LOO (2-1)

LOO· + L-H → L· + L-OOH(2-2)

L-OOH + Fe^2+^ → LO· + OH^−^ + Fe^3+^ (2-3)

LOO· + LOO· → Nonradical breakdown products(3)

GPX4 circulates between oxidation and reduced states, thereby eliminating excess reactive oxygen through a redox reaction [75]. The catalytic cycle is divided into two phases of a mutual cycle according to the ping-pong mechanism [76], as shown in the formulae below. In the first stage, selenium alcohol in GPX4 is oxidized into selenium acid by hydrogen peroxide (ROOH). Simultaneously, a molecule of GSH and selenium substitute acid form selenium sulfide, and oxygen is removed as water. Correspondingly, another molecule of GSH is reduced to selenium sulfate through the thiol-disulfide exchange to produce GSSG. In the second stage, glutathione reductase (GSR) uses NADPH to reduce GSSG to GSH [17,77]. 

The equations of the catalytic cycle of GPX4:

GPX4-Se^−^ + L-OOH → GPX4-SeOH + L-OH

GPX4-SeO^−^ + H+ +GSH → GPX4-Se-SG + H_2_O

GPX4-Se-SG + GSH → GPX4-Se + H + GSSG

GSSG + NADPH + GSR → NADP^+^ + GSH

## 6. GPX4 and Ferroptosis

**Figure 1:**
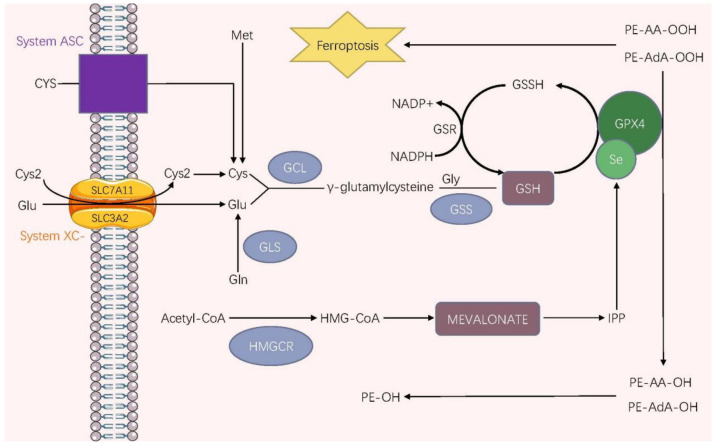
The ferroptosis regulatory pathway correlated to GPX4. Under oxidative conditions, the most upstream event of ferroptosis is the importation of Cys2 via system XC-. Cys2 is then reduced to Cys, which the system ASC can directly take under reduced conditions after it has been imported. In addition, there is another source of intracellular Cys in which Met is converted into Cys through the transsulfuration pathway. GSH, a vital antioxidant, is created by Cys, Glu, and Gly under two catalysis steps involving GCL and GSS cytosolic enzymes. GPX4 accepts electrons from GSH molecules, which reduces the toxic PE-AA-OOH/PE-AdA-OOH into nontoxic PE-AA-OH/PE-AdA-OH, ultimately inhibiting ferroptosis. GSH, the electron donor, is oxidized to GSSG that is then reduced to GSH by NADPH under the reductase GSR. Abbreviations: Gln, glutamine; GLS, glutaminase; Se, selenium; HMGCR, 3-hydroxy-3-methylglutaryl-CoA reductase; HMG-CoA, 3-hydroxy-3-methylglutaryl-coenzyme A; IPP, isopentenyl pyrophosphate; NADP+, nicotinamide adenine dinucleotide phosphate; PE-OOH, phosphatidyl-ethanolamine hydroperoxide; PE-OH, phosphatidyl-ethanolamine hydroxide.

Ferroptosis is a novel form of iron-dependent cell death characterized by excessive iron accumulation and lipid peroxidation and is morphologically, genetically, and biochemically distinct from other known cell death processes, such as apoptosis, necrosis, and autophagy. GPX4 catalyzes the conversion from R-OOH into R-OH, thereby preventing iron-dependent lipid reactive oxygen production and inhibiting ferroptosis. Therefore, GPX4 is a key regulator of ferroptosis (characterized by lipid peroxidation) and depends on iron and ROS. GPX4 participates in forming the system x_c_^−^/GSH/GPX4 axis, which combats cellular phospholipid peroxidation, thereby inhibiting ferroptosis [6,10,78] [79]. System X_c_^−^, a disulfide-linked heterodimer composed of the catalytic subunit solute carrier family 7 member 11 (SLC7A11) and the regulatory subunit solute carrier family 3 member 2 (SLC3A2) visa disulfide bond, exchanges cysteine with glutamate (Glu) across the plasma membrane at 1:1 mole ratio [80,81,82,83,84]. After being transported in, cystine transforms into cysteine, and another source of cysteine involves the reverse transsulfuration of methionine (Met) [83]. In addition, the alanine-serine-cysteine (ASC) translator can directly transport cysteine into cells at reduced extracellular conditions [85]. Cysteine and glutamate are synthesized into γ-glutamylcysteine under the action of glutamate-cysteine ligase (GCL). Then GSH is formed by γ-glutamylcysteine and glycine (Gly) under the activity of glutathione synthetase (GSS) [86]. Glutathione is essential for GPX4 to manifest its protective effect of guarding cells against ferroptosis. Bimolecular GSH provides electrons to GPX4 to reduce cellular toxic phospholipid hydroperoxides (PE-AdA-OOH, PE-AA-OOH) into nontoxic phospholipid alcohols (PE–AdA–OH, PE–AA–OH), thereby interrupting the chain reaction of lipid peroxidation and exerting the surprising functions of suppressing ferroptosis [83]. The MVA pathway has been regarded as a vital mechanism of selenoprotein synthesis. In the MVA pathway, acetyl-CoA is transformed into HMG-CoA under 3-hydroxy-3-methylglutaryl-CoA reductase (HMGCR), which is then reduced to mevalonate that is conversely converted to IPP [87]. Consequently, with the help of IPP, a Sec residue can be added to the catalytic center of GPX4, one which contributes to the activation of GPX4 and the inhibition of ferroptosis [48,88]. Moreover, IPP generates coenzyme Q10 (CoQ10), which subsequently enters into the ferroptosis suppressor protein 1 (FSP1) pathways to induce ferroptosis in cooperation with FSP1 [76] (See Figure 1).

In The Cancer Genome Project (TCGA) database, several researchers have reported that the expression of GPX4 is higher in various tumors than in normal tissues, including kidney chromophobe (KICH) [89], prostate adenocarcinoma (PRAD) [90], thyroid carcinoma (THCA) [91], colon adenocarcinoma (COAD) [92], kidney renal clear cell carcinoma (KIRC), cervical and endocervical cancer (CESC), lung adenocarcinoma (LUAD) [93], and rectum adenocarcinoma (READ). The expression of GPX4 is generally higher in patients with multiple cancers than in normal tissues. This suggests that the ultimate identity of GPX4 could be an unconfirmed oncogene causing harm to patients with various cancers [94]. Some researchers have discovered the four ways of initiating ferroptosis [95]: Class I ferroptosis inducers work by driving depletion of GSH; Class II ferroptosis inducers act by directly targeting and inactivating GPX4; Class III ferroptosis inducers cause depletion of GPX4 and CoQ10 generated by the MVA pathway, and Class IV ferroptosis inducers increase the LIP or oxidize iron to induce lipid peroxidation [96]. It should be noted that the first three classes of ferroptosis inducers are strongly associated with GPX4, which indicates the vast potential of GPX4 inhibiting cancers via ferroptosis. Although interruption of GSH may indirectly inactivate GPX4, this mechanism is less efficient in vivo due to other compensatory pathways. In contrast, strategies that directly target GPX4 can be more effective in inducing ferroptosis both in vitro and in vivo [97]. 

## 7. Potential Role of GPX4 in Cancer Therapy

Acquired drug resistance is one of the significant limitations which blocks targeted anticancer therapies from achieving better results on stability and integrity [98]. Non-mutational drug-tolerant cells can constitute reservoirs where fully drug-resistant cells are formed, ultimately leading to tumor recurrence [99,100,101]. Interestingly, the loss of GPX4’s function contributes to the selective ferroptosis dependency of cells and tumor relapse, which has been shown in non-small cell lung cancer, pancreatic cancer, prostate cancer, and melanoma cells [102]. Cancer cells with a high mesenchymal state have higher clinical stages and are selectively sensitive to ferroptosis, which suggests that they are sensitive to GPX4 inhibition [103]. Several studies have revealed that ionizing radiation (IR) causes ferroptosis in fibrosarcoma, breast cancer, renal carcinoma, and esophageal adenocarcinoma [104]. In addition, ferroptosis inducers targeting GPX4 have been found to enhance the sensitivity of radiotherapy in xenograft models [104]. Treatment with doxorubicin significantly reduced the tumor mass composed of HCT116 (a human colon cancer cell line) cells with *gpx4* knockdown compared to the control nude mice without *gpx4* knockdown [105]. As an efficient inhibitor of ferroptosis, GPX4 has provided drug resistance to tumor therapies, which is a significant challenge in the clinic with novel molecular targets and therapeutic strategies [106,107].

To confirm the role of GPX4 in tumorigenesis, Schneider et.al. hypothesized that GPX4 played a putative regulatory role during tumor progression and conducted an experiment to inactivate GPX4 in murine embryonic fibroblasts (MEFs) [108]. GPX4 inactivation in vitro caused instantaneous cell death. Surprisingly, transformed *GPX4^+/−^* survived in Matrigel and produced tumor spheroids. The tumor cells were implanted subcutaneously in mice. Later, a tumor was harvested with a similar volume and weight as wild-type tumors but a strong vascular phenotype—an increase in microvessel density and a reduction in large diameter vessels. Inhibiting 12/15-LOX pharmacologically reversed the phenotype, bringing vessel morphology to normal. Thus, it was concluded that GPX4 is a key regulator of tumor progression and vessel maturation through 12/15-LOX activity control.

**Figure 2:**
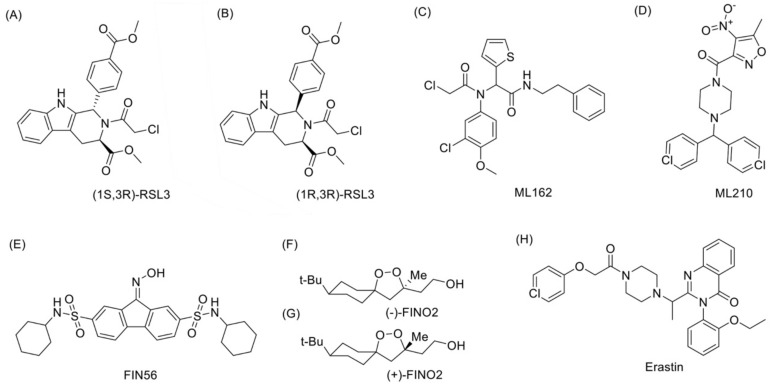
Structures of GPX4-related molecules mentioned in the article. (**A**) (1S-3R)-RSL3, or RSL3, the first described Class II inhibitor that directly targets GPX4 to trigger ferroptosis. (**B**) (1R-3R)-RSL3, a diastereomer of (1S-3R)-RSL3, which loses its HRAS selectivity. (**C**,**D**) ML162 and ML 210, the direct GPX4 inhibitors. (**E**) FIN56, a Class III ferroptosis inducer, mainly triggers ferroptosis through cellular depletion of GPX4. (**F**,**G**) FINO2, a Class IV inducer that does not directly inhibit GPX4. (**H**) Erastin, a classic Class I inducer that function by inhibiting system x_c_^−^ instead of affecting GPX4 directly.

To date, there are no published reports of GPX4-relevant drugs under clinical trial. Thus, we searched for known GPX4 inhibitors that can trigger ferroptosis (Figure 2), intending to provide insights for drug development. While the significance of selenium to GPX4 in cancer therapy has not yet been explored, common approaches to GPX4-relevant cancer therapy involve downregulating GPX4-induced antioxidation, a well-known method of activating ferroptosis. Studies have proposed that most cancer cells are under high levels of oxidative stress [109], which needs to be counteracted by increasing the ROS-scavenging capacity to prevent oxidative damage [110]. 

RSL3 was the first described Class II inhibitor that directly targets GPX4 to trigger ferroptosis [80]. Previous affinity-based chemo proteomics showed that the chloroacetamide moiety in the RSL3 structure is necessary for its activity. RSL3 targets enzymes (especially Sec) with a nucleophilic site and directly inactivates GPX4 by alkylating its Sec residue [111]. Among the four diastereomers of RSL3, only (1S, 3R)-RSL3 showed a much more effective selective lethality to the HRAS-containing BJeLR cells system. This may be attributed to binding (1S, 3R)-RSL3 to one or more proteins in HRAS-expressed BJ cells [10]. Fluorescein tag can be linked with (1S, 3R)-RSL3 to the phenyl substituent via a polyethylene glycol (PEG) linker. The oncogenic HRAS selectivity of RSL3 differs among its diastereomers, as (1R, 3R)-RSL3 loses HRAS selectivity [83]. Other direct GPX4 inhibitors include ML162, ML210, and a series of DPI compounds [112]. In a previous study, the direct GPX4 inhibitors were tested in vitro, and positive results were harvested. However, no suitable inhibitor was found among them for in vivo application due to their poor solubility and hard characterization of their pharmacokinetics [95]. 

Ferroptosis inducer 56 (FIN56), a Class III ferroptosis inducer, mainly triggers ferroptosis through cellular depletion of GPX4 [95]. An assay of chemo proteomics revealed that FIN56 is associated with the mevalonate pathway via a GPX4-independent manner. In the MVA pathway, FIN56 binds to and subsequently activates squalene synthase, a participant of cholesterol synthesis, and then induces depletion of CoQ10 that functions as an endogenous lipophilic antioxidant with FSP1 [113].

Notably, 1,2-dioxolane FINO2, a Class IV inducer, does not directly inhibit GPX4 [114,115]. FINO2 has been shown to effectively oxidize ferrous iron independent of ALOX activity in vitro, thereby promoting the Fenton reaction, which results in more wide-ranging oxidized phospholipids than those induced by GPX4 inhibition [115]. When treating cells with FINO2, the iron chelator (deferoxamine) exerted a protective effect. Interestingly, expression of iron transition protein (TFR1, transferrin receptor 1), regulatory (IREB2, iron-responsive element-binding protein 2), and storage proteins (FTL1, ferritin light chain 1) remained unchanged, suggesting a critical involvement of the cellular labile iron pool [80]. 

Evidence suggests that Class I inducers do not affect GPX4 directly. Instead, they function by inhibiting system x_c_^−^ to reduce cystine uptake and prevent conversion to cysteine, which contributes to GSH synthesis [88], the essential substrate for GPX4 activity. GSH, a cofactor and reaction substrate for GPX4, is vital due to its lipid repair function. Depleting GSH through cysteine starvation causes loss of GPX4 activity, resulting in the accumulation of unrepaired lipid peroxides and ferroptosis [95]. Erastin and glutamate, the system x_c_^−^ inhibitors, were inducers of ferroptosis [6]. Subsequent studies identified more inhibitors, including erastin analogs (imidazole ketone erastin (IKE) and piperazine erastin (PE)) and previously developed sorafenib and sulfasalazine [116]. All these are suitable for in vitro use, but they should be used with care since sorafenib and glutamate also activate other non-ferroptotic mechanisms. However, erastin has low solubility, unsatisfying pharmacokinetics, and poor metabolic stability, limiting its in vivo application [95]. Recent studies suggested that applying erastin in combination therapy may be a practical approach. For example, Zhu et al. [117] constructed a carrier-free nano-drug containing erastin and photosensitizer Ce6. The drug exhibited better solubility and metabolic stability and improved the curative effect when applied along with laser treatment in a mice experiment treating CAL-27 tumor cells. 

## 8. Conclusions and Perspectives

GPX4, as the only GPX family member with membrane lipid removal product of hydrogen peroxide, has a unique antioxidant function, which is dependent on the presence of selenoproteins (with Sec as the essential active site). GPX4 has an excellent antioxidant capacity in inhibiting ferroptosis. Several studies have revealed that the expression of GPX4 in many cancer cells is higher than in normal tissues, which enhances ferroptosis resistance. This suggests that inhibition of the GPX4 pathway to induce cell ferroptosis could theoretically be an effective method of cancer therapy. However, research on improving the pharmacokinetic properties of the inhibitors is still ongoing, which is essential to achieve a better clinical application. Currently, new GPX4-related drugs have yet to be developed, and research combined with the development of nano-drugs has not yet been conducted, all of which has bright prospects in the future.
